# The Impact of Testicular Prosthesis on Cancer Patients’ Intimacy and Quality of Sex Life

**DOI:** 10.7759/cureus.93935

**Published:** 2025-10-06

**Authors:** Hamid El Kaddouri, Spyridon Sideris, Jeanne Beirnaert, Michael Reich, Jeremy Blanc, Raquel Da Silva Maia, Myky Nguyen, Nieves Martinez Chanza, Thierry Roumeguere

**Affiliations:** 1 Urology, Hôpital Universitaire de Bruxelles, Brussels, BEL; 2 Oncology, Hôpital Universitaire de Bruxelles, Brussels, BEL; 3 Ophthalmology, Eye Center at Medical Center, University of Freiburg, Freiburg, DEU

**Keywords:** implants, prostheses, quality of life, sexuality, testicular neoplasms

## Abstract

Introduction

Post-orchiectomy testicular prosthesis (TP) implantation is offered to patients to minimize psychological distress. Sexual dysfunction, negative body image perception, and fertility outcomes are well addressed in the literature; however, little is known about the impact of TP on sexual life.

Methods

This is a retrospective, single-center cohort study conducted at Hôpital Universitaire de Bruxelles. Patients with TP completed a questionnaire exploring body image perception, reasons for TP acceptance, overall satisfaction, and both short- and long-term impacts on their sexual life and quality of life. Statistical analyses using the Chi-square and Wilcoxon tests were performed to correlate TP satisfaction with patients’ characteristics, sexual dysfunction, fertility, and interpersonal or professional stability.

Results

Sixty patients were included between 2000 and 2021, with a median follow-up of 6.2 years (range: 3.4-11.7). The mean age at orchiectomy was 32 years (range: 17-49). During this period, 10 (17%) patients required testosterone supplementation, 10 (18%) developed depression, 17 (31%) changed their profession, 9 (16%) separated from their partner, and 15 (30%) fathered at least one child. Weight gain was observed in 40 (67%) patients. Forty-eight (80%) patients were satisfied with their TP. However, 24 (40%) felt it was too high, 31 (52%) too firm, and 12 (20%) too large. There was no significant impact on overall sexual performance (*p* > 0.05), although erectile dysfunction was significantly associated with dissatisfaction with the prosthesis (*p* = 0.01).

Conclusions

Overall TP satisfaction was high, with no negative impact on patients’ sexual life or quality of life. Patients who were dissatisfied with the TP were predominantly those who experienced erectile dysfunction.

## Introduction

Testicular cancer (TC) is among the most frequent solid malignant tumors in young adult males, with an increasing incidence noted in Europe [1,2]. TC has a high relative five-year survival rate of 99% for localized disease in the studied populations [3].

Orchiectomy is the standard of care for first-line treatment of TC. However, orchiectomy can lead to a perceived impairment in body appearance in approximately 25% of testicular tumor survivors, as well as a fear of loss of masculinity [4]. This may have a considerable impact on patients’ sexual life, especially among younger individuals [5]. Testicular prosthesis (TP) placement is offered to patients following orchiectomy to minimize psychological distress and to help restore quality of life and self-esteem [6]. Surprisingly, very few studies have evaluated the impact of TP on the daily lives of TC survivors [7].

Previous studies have mainly focused on body image and psychological distress following orchiectomy, highlighting feelings of loss, reduced masculinity, and altered self-perception [4,5]. These aspects are well documented and have driven the rationale for TP placement. However, data remain scarce and fragmented regarding the impact of TP on intimacy, sexual satisfaction, and the quality of sexual life in TC survivors. Recent reviews emphasize that while body image outcomes are relatively well explored, a gap persists in understanding how TP influences domains such as sexual desire, erectile function, and partner intimacy [6,7]. Addressing this gap is critical, as sexual quality of life is a key component of survivorship. The present study contributes to this field by specifically assessing both short- and long-term effects of TP on sexual response, intimacy, and overall quality of life in a single-center cohort.

We aimed to investigate the impact of TP on patients’ quality of life and sexual satisfaction.

The primary outcome of this study was to determine the impact of TP on TC patients’ sexual life (sexual desire, erection, ejaculation, frequency and quality of sexual encounters, and intimacy) in the short term (≤1 year after TP) as well as in the long term (>1 year after TP).

The secondary objectives were to establish correlations between TP satisfaction and several clinical and psychosocial factors, including TC histology, stage and prognosis group classification, age at diagnosis, adjuvant therapy, cancer treatment, testosterone supplementation, body weight modification, depression, and professional or interpersonal (relationship) stability. In addition, the study aimed to evaluate the association between sexual response (desire, erection, and ejaculation) and factors such as TC histology, age at diagnosis, cancer treatment type, body weight modification, psychological issues, professional changes, relationship stability, need for testosterone supplementation, paternity rate, and the quality of sexual encounters in both the short- and long-term periods following TP. Furthermore, the study sought to assess the impact of professional change and depression on the frequency and quality of sexual encounters among patients after TP placement.

## Materials and methods

Study design and participants

This is a retrospective, single-center cohort study conducted at the Hôpital Universitaire de Bruxelles. Sexually active adult males, defined as those engaging in sexual activity at least once in the preceding six months, with histologically confirmed testicular cancer (germ cell carcinoma) who underwent orchidectomy and subsequent testicular prosthesis placement between 2000 and 2021 were eligible for inclusion, following approval by the local ethics committee (reference: CE2146).

Materials and data collection

Eligible patients were offered an original structured questionnaire (Appendix 1) after providing written informed consent. The questionnaire was designed by the study investigators following a review of relevant literature and discussions within a panel of urologists experienced in testicular cancer and prosthesis implantation. Although it was not a formally validated instrument, its items were adapted from surveys previously used in studies assessing outcomes of testicular prosthesis [7]. To ensure clarity and face validity, the questionnaire was pilot tested in a small group of patients (n = 5) before distribution, and minor wording adjustments were made accordingly. The final version of the questionnaire comprised 18 questions divided into five parts (A-E). Part A focused on the reason for acceptance of the testicular prosthesis (TP), while Part B included a general questionnaire evaluating patients’ overall satisfaction with their TP. Part C explored the effects of the TP on current sexual life, including possible dysfunctions in sexual desire, erection, and ejaculation. Part D examined the impact of the TP on the frequency and quality of sexual encounters, as well as its influence on intimacy within one year after TP placement. Part E addressed the same domains as Part D but for a period exceeding one year after TP. The questionnaire was distributed via email using the REDCap system (Research Electronic Data Capture).

Statistical analysis

Statistical analyses were performed using SAS version 9.4 (SAS Institute Inc., Cary, NC, USA). Quantitative variables are presented as medians with IQRs, and qualitative variables as counts and percentages. The Chi-square (χ²) test was used to compare qualitative variables, while the Wilcoxon test was applied to quantitative variables. To assess correlations between quantitative variables, the Spearman correlation test was used. Probability (p) values < 0.05 were considered statistically significant.

## Results

Sixty patients were included in the study. Patients’ characteristics and evolution during follow-up are summarized in Table 1. The mean age at orchiectomy was 32 years (range: 17-49 years). The median follow-up period was 6.2 years (range: 3.4-11.7 years).

**Table 1:** Patients’ characteristics. Note: n = number of patients; BEP = adjuvant Bleomycin, Etoposide, and Cisplatin.

Characteristic	Subgroup	Value
Age at orchidectomy, years ± SD	Mean	32 ± 7
	Range (min-max)	17-49
Histology, n (%)	Seminoma	28 (47)
	Non-seminoma	32 (53)
Prognostic group, n (%)	Good	56 (93)
	Intermediate	3 (5)
	Poor	1 (2)
Stage, n (%)	I	34 (57)
	II	19 (32)
	III	7 (12)
Chemotherapy, n (%)	Yes	44 (73)
	No	16 (27)
Lines of chemotherapy, n (%)	0	16 (27)
	1	42 (70)
	2	2 (3)
Type of chemotherapy, n (%)	BEP	26 (59)
	Carboplatin	18 (41)
	Not available	16
Radiotherapy, n (%)	Yes	2 (3)
	No	57 (97)
	Not available	1
Surgery for residual disease, n (%)	Yes	9 (15)
	No	50 (85)
	Not available	1
Paternity after orchidectomy, n (%)	Yes	15 (30)
	No	35 (70)
	Not available	10
Depression, n (%)	Yes	10 (18)
	No	45 (82)
	Not available	5
Professional change, n (%)	Yes	17 (31)
	No	37 (69)
	Not available	6
Separation, n (%)	Yes	9 (16)
	No	46 (84)
	Not available	5
Testosterone supplementation, n (%)	Yes	10 (17)
	No	49 (83)
	Not available	1
Weight change between orchidectomy and last follow-up, n (%)	Weight gain (>0)	40 (67)
	Stable (=0)	10 (17)
	Weight loss (<0)	10 (17)
Follow-up, years ± SD (median)	Mean	7.7 ± 5.5 (6.2)

In terms of body modifications, weight gain was observed in 40 (67%) patients, with a median gain of 5.5 kg (range: 3-13 kg).

Results of the survey are reported in Table 2.

**Table 2:** Survey results.

Question	Answer Yes n (%)	Answer No n (%)	Answer “I Don’t Know” n (%)
A. Decision-making factors			
1. You agreed to the implantation of a testicular prosthesis. Is the “normal” appearance of the organ of high importance to you?	47 (78)	10 (17)	3 (5)
2. Did you have a steady partner when you decided to have a testicular prosthesis?	46 (77)	14 (23)	0 (0)
3. Did having or not having a partner play a role in your decision?	7 (12)	52 (87)	1 (1)
B. Effect on sexual life			
1. Do you find that your testicular prosthesis negatively affects your sexual desire (libido)?	1 (2)	56 (93)	3 (5)
2. Do you find that having a testicular prosthesis negatively affects the quality of your erections?	3 (5)	51 (85)	6 (10)
3. Do you find that you have problems with ejaculation due to the testicular prosthesis?	5 (8)	50 (84)	5 (8)
C. Short-term effect (period less than 1 year after testicular prosthesis insertion)			
1. Did having a testicular prosthesis decrease the frequency of sexual intercourse?	2 (3)	51 (85)	7 (12)
2. Have you started testosterone replacement therapy?	8 (13)	51 (85)	1 (2)
3. Did having a testicular prosthesis negatively affect the quality of sexual intercourse?	5 (8)	51 (85)	4 (7)
4. Do you think the testicular prosthesis had an impact on your intimacy?	4 (7)	50 (83)	6 (10)
D. Long-term effect (period more than 1 year after testicular prosthesis insertion)			
1. Did having a testicular prosthesis decrease the frequency of sexual intercourse?	1 (2)	55 (91)	4 (7)
2. Have you started testosterone replacement therapy?	8 (13)	51 (85)	1 (2)
3. Did having a testicular prosthesis negatively affect the quality of sexual intercourse?	4 (7)	52 (86)	4 (7)
4. Do you think the testicular prosthesis had an impact on your intimacy?	4 (7)	52 (86)	4 (7)

Decision-making factors for TP placement (A)

Among the patients, 78% considered the normal appearance of genital organs important, but only 12% felt that having a stable partner influenced their decision. No statistical correlation was found (p > 0.05) between having a partner and TP satisfaction.

Impact of TP placement on patients’ sexual response (B)

Seventy-eight percent of patients reported no issues with libido, erection, or ejaculation, while 22% admitted at least one sexual disorder or were uncertain. Only 4% reported multiple sexual dysfunctions.

Short-term impact of TP placement on patients’ sexual life (C)

Within one year after TP, 85% of patients reported no negative impact on sexual life, frequency, quality of sexual encounters, or intimacy with partners.

Long-term impact of TP placement on patients’ sexual life (D)

After at least one year, over 86% of patients reported no negative impact of TP on sexual life, frequency, quality, or intimacy. Only five patients experienced reduced frequency or quality of sexual encounters.

Results of the survey on satisfaction with TP placement are reported in Tables 3.

**Table 3:** Results on satisfaction with TP. TP: Testicular prosthesis.

Question	Answer Yes n (%)	Answer No n (%)	Answer “I Don’t Know” n (%)
Are you satisfied overall with your testicular prosthesis?	48 (80)	6 (10)	6 (10)

Overall, 80% of patients reported being satisfied with their TP. However, when satisfaction was analyzed across the three specific domains (position, consistency, and size), only 23% of patients considered all three aspects satisfactory (Tables 4-6).

**Table 4:** Results on TP position. TP: Testicular prosthesis.

Question	Answer Very High n (%)	Answer Appropriate n (%)	Answer Very Low n (%)
You find that the position of the prosthesis is:	24 (40)	34 (57)	2 (3)

**Table 5:** Results on TP consistency. TP: Testicular prosthesis.

Question	Answer Too Firm n (%)	Answer Appropriate n (%)	Answer Too Soft n (%)
You find that the consistency of the prosthesis is:	31 (52)	27 (45)	2 (3)

**Table 6:** Results on TP size. TP: Testicular prosthesis.

Question	Answer Too Small n (%)	Answer Appropriate n (%)	Answer Too Big n (%)
You find that the size of the prosthesis is:	4 (7)	44 (73)	12 (20)

Impact of TP on sexual life

Results are reported in Tables 7-8. There was no significant negative impact of TP on sexual response, as the majority of patients reported preserved libido (96% among satisfied patients vs. 67% among dissatisfied, p = 0.09), normal erections (92% vs. 50%, p = 0.01), and normal ejaculation (83% vs. 67%, p = 0.55). Dissatisfaction with TP was thus strongly correlated with erectile dysfunction, whereas libido and ejaculation were not significantly affected. In the short term, most patients reported no decrease in the frequency (85% vs. 83%, p = 0.65) or quality (90% vs. 50%, p = 0.06) of sexual intercourse, and no impact on intimacy (85% vs. 67%, p = 0.28). Similarly, in the long-term follow-up, over 90% of patients across groups reported preserved frequency, quality, and intimacy (all p > 0.12).

**Table 7:** Correlation of overall TP satisfaction with short- and long-term impact of TP. Note: The Chi-square (χ²) test was used for all categorical variables. TP: Testicular prosthesis.

Question	p-value	Yes (n=48)		No (n=6)		I Don’t Know (n=6)	
		n	%	n	%	n	%
C. Effect on sexual life							
Do you find that your testicular prosthesis negatively affects your sexual desire (libido)?	0.09						
Yes		1	2	-	-	-	-
No		46	96	4	67	6	100
I don’t know		1	2	2	33	-	-
Do you find that having a testicular prosthesis negatively affects the quality of your erections?	0.01*						
Yes		1	2	2	33	-	-
No		44	92	3	50	4	67
I don’t know		3	6	1	17	2	33
Do you find that you have problems with ejaculation due to the testicular prosthesis?	0.55						
Yes		4	8	1	17	-	-
No		40	83	4	67	6	100
I don’t know		4	8	1	17	-	-
D. Short-term effect							
Did having a testicular prosthesis decrease the frequency of sexual intercourse?	0.65						
Yes		2	4	-	-	-	-
No		41	85	5	83	5	83
I don’t know		5	10	1	17	1	17
Have you started testosterone replacement therapy?	0.86						
Yes		7	15	1	17	-	-
No		40	83	5	83	6	100
I don’t know		1	2	-	-	-	-
Did having a testicular prosthesis negatively affect the quality of sexual intercourse?	0.06						
Yes		3	6	2	33	-	-
No		43	90	3	50	5	83
I don’t know		2	4	1	17	1	17
Do you think the testicular prosthesis had an impact on your intimacy?	0.28						
Yes		2	4	1	17	1	17
No		41	85	4	67	5	83
I don’t know		5	10	1	17	-	-
E. Long-term effect	0.69						
Did having a testicular prosthesis decrease the frequency of sexual intercourse?							
Yes		1	2	-	-	-	-
No		44	92	5	83	6	100
I don’t know		3	6	1	17	-	-
Have you started testosterone replacement therapy?	0.19						
Yes		7	15	1	17	-	-
No		41	85	4	67	6	100
I don’t know		-	-	1	17	-	-
Did having a testicular prosthesis negatively affect the quality of sexual intercourse?	0.25						
Yes		3	6	1	17	-	-
No		43	90	4	67	5	83
I don’t know		2	4	1	17	1	17
Do you think the testicular prosthesis had an impact on your intimacy?	0.12						
Yes		3	6	-	-	1	17
No		43	90	4	67	5	83
I don’t know		2	4	2	33	-	-

**Table 8:** Correlation of overall TP satisfaction with patients’ characteristics. N: Number of patients; Q1-Q3: First quartile to third quartile; BEP: Adjuvant Bleomycin, Etoposide, and Cisplatin. Statistical test: Chi-square (χ²) test was used for categorical variables, and the Wilcoxon rank-sum test was used for continuous variables (age).

	Are you satisfied overall with your testicular prosthesis?												P-value
	Yes (n=48)				No (n=6)					I don’t know (n=6)			
	n	%			n		%			n		%	
Age at orchidectomy													0.06
Median (Q1 to Q3)	30.5 (27-35)			37.5 (34-38)					34.5 (32-36)				
Histology													0.32
Seminoma	20		42	4		67		4			67		
Non seminoma	28		58	2		33		2			33		
Prognostic group													0.09
Good	46		96	4		67		6			100		
Intermediate	1		2	2		33		-			-		
Poor	1		2	-		-		-			-		
Stage													0.53
1	27		56	2		33		5			83		
2	15		31	3		50		1			17		
3	6		13	1		17		-			-		
Chemo													1
Yes	35		73	5		83		4			67		
No	13		17	1		17		2			33		
Type chemo													0.39
BEP	22		63	3		60		1			25		
Carboplatin	13		37	2		40		3			75		
Not applicable	13			1				2					
Radiotherapy													1
Yes	2		4	-		-		-			-		
No	46		96	6		100		5			83		
Not available	-			-				1					
Surgery residual disease													0.41
Yes	6		13	2		33		1			17		
No	41		87	4		67		5			83		
Not applicable	1												
Paternity after orchidectomy													1
Yes	13		33	1		20		1			20		
No	27		68	4		80		4			80		
Not available	8			1				1					
Depression													0.24
Yes	10		23	-		-		-			-		
No	33		77	6		100		6			100		
Not available	5												
Profession change													0.23
Yes	16		36	-		-		1			20		
No	28		64	5		100		4			80		
Not available	4			1				1					
Separation													0.82
Yes	8		18	1		20		-			-		
No	37		82	4		80		5			83		
Not available	3			1				1					
Testosterone supplementation													0.82
Yes	9		19	1		17		-			-		
No	38		81	5		83		6			100		
Not available	1												
Weight change													0.23
Gain	33		69	3		50		4			67		
Stable	9		19	-		-		1			17		
Loss	6		13	3		50		1			17		

Regarding patient characteristics, younger patients (median age 30.5 years) tended to be more satisfied than older patients (median 37.5 years), although this trend did not reach statistical significance (p = 0.06). Patients with a good prognostic group classification also showed higher satisfaction (96% vs. 67%, p = 0.09). No significant associations were observed between TP satisfaction and histology, stage, type of chemotherapy, radiotherapy, residual disease surgery, testosterone supplementation, or relationship status (all p > 0.2).

Body-weight changes were frequent, with 67% of patients reporting weight gain, but these modifications did not influence TP satisfaction (p = 0.23) or sexual frequency and quality. Similarly, 18% of patients reported depression and 31% reported professional changes, but neither factor was associated with reduced satisfaction or impaired sexual activity (p > 0.2). Finally, only a small subset of patients (5%) reported a decline in erection quality, which was not linked to depression or job changes.

Regarding quantitative variables, the Wilcoxon rank-sum test was applied. Patients satisfied with their TP tended to be younger at the time of orchidectomy compared with dissatisfied patients (median 30.5 vs. 37.5 years; Z = -1.87, p = 0.06). Follow-up duration did not differ significantly between groups (median 6.2 vs. 7.5 years; Z = -0.74, p = 0.46) (Table 9).

**Table 9:** Wilcoxon rank-sum test results for quantitative variables.

Variable	Satisfied (n=48) Median (Q1-Q3)	Dissatisfied (n=6) Median (Q1-Q3)	Wilcoxon Z	p-value
Age at orchidectomy (years)	30.5 (27-35)	37.5 (34-38)	-1.87	0.06
Follow-up (years)	6.2 (3.4-11.7)	7.5 (5.2-10.3)	-0.74	0.46

## Discussion

Testicular prosthesis (TP) placement is offered to improve body image perception and aims to enhance the quality of life following orchidectomy [8]. While Adshead J et al. [9] reported lower acceptance rates among married or stably partnered men, our study did not reveal a statistically significant correlation between relationship status and TP satisfaction (p > 0.2), potentially reflecting differences in population characteristics or study design. Other series have shown that the likelihood of accepting a TP decreases with age and with the probability of living in a steady relationship [10]. Although the desire for a normal genital appearance was the primary reason for TP placement, this motivation does not appear to be influenced by the presence or absence of a consistent partner at the time of decision-making. These findings align with results reported by Xylinas E et al. [11], with 96% of patients indicating that they would undergo the procedure again, regardless of their relationship status.

Patients’ attitudes toward TP are generally positive, and satisfaction rates exceed 70% across studies [12]. Appropriate size and position have been statistically correlated with overall TP satisfaction [13]. In accordance with the existing literature, the main TP issues identified were a position that was too high, consistency that was too firm, and size that was too large. In our study, no statistically significant associations were found between these factors and TP satisfaction (all p > 0.2), suggesting that their potential role should be examined further in larger and prospective studies. This underscores the importance of addressing mental health and hormonal balance in patients undergoing TP, which could represent valuable areas for future research.

Sexual dysfunction is often experienced by TC survivors. Several reports have evaluated erectile function in this specific population [14-16]. A multicenter study involving more than 1,200 survivors reported a 4.2-fold higher risk of erectile dysfunction in testicular tumor survivors compared to controls. Approximately one-third of testicular tumor survivors also experienced ejaculatory dysfunction [17].

Sexual activity after TP is poorly discussed in the literature. The few studies analyzing this aspect used simple and generic questions [18,19]. Other aspects of sexual activity, such as the frequency and quality of sexual encounters, are rarely discussed in the literature, particularly for TC survivors with a TP.

In the present study, there was no negative effect of TP on patients’ sexual response (desire, erection, ejaculation). However, dissatisfaction with TP was correlated only with low-quality erections. The frequency and quality of sexual encounters were not affected by TP in either the short-term or long-term follow-up periods.

Few studies have specifically assessed the topic of romantic relationships in testicular cancer (TC) survivors. Patients in committed romantic relationships at the time of diagnosis coped much better with the treatment and reported increased closeness with their partners [20-23]. There is also evidence suggesting that, for a subgroup of survivors, TC exacerbated pre-existing relationship conflicts or created new ones, leading to relationship dissolution [24]. Additionally, unpartnered TC survivors reported worse satisfaction with erection and orgasm and lower overall sexual satisfaction compared with partnered survivors, despite higher levels of sexual desire [25,26]. To our knowledge, the specific role of TP in patients’ intimacy and romantic relationships has not been discussed in the literature.

Sixteen percent of patients experienced separation from their partners in the years following TP, which is consistent with existing literature. However, separation was not statistically correlated with overall TP satisfaction.

Qualitative studies indicate that more than half of TC survivors face infertility issues. A meta-analysis of 12 cohort studies revealed an increased risk of testosterone deficiency among TC patients [27]. Despite the presence of hypogonadism, many survivors are able to father children after completing treatment, although the process can be prolonged. Androgen deficiency is known to impact spermatogenesis and libido by reducing the stimulation of Sertoli cells and altering the hypothalamic-pituitary-gonadal axis, potentially complicating the reproductive process even in patients who maintain fertility [28]. In this study, 18% of patients required testosterone supplementation after TP, and 30% of the cohort achieved parenthood. These findings align with existing literature; however, testosterone supplementation and paternity were not statistically associated with overall satisfaction.

Another issue that emerged was the relationship between professional changes, depression, and their potential impact on the frequency and quality of sexual encounters. While data on job change and depression were available for most patients, the analysis indicated that neither professional change nor depression had a negative influence on sexual encounters. This suggests that, contrary to what might be expected, these factors did not significantly affect the frequency or quality of sexual activity.

According to the findings of the Platinum Study, which investigated 1,214 TC survivors, the most frequent adverse outcome observed at a median of 4.2 years after chemotherapy completion was obesity, with a prevalence of 71.5% [29]. A greater risk for metabolic syndrome was also found in patients receiving chemotherapy compared with those who underwent surgery alone [30]. In the existing literature, no studies have reported a correlation between body weight modification and TP. In the present study, although more than half of the patients experienced weight gain after TP, body weight modifications did not influence the frequency or quality of sexual encounters in either the short- or long-term period, and no statistically significant correlation was observed with TP satisfaction.

Limitations of our study include the small sample size and the absence of a control group of patients who underwent orchiectomy without TP, which would have provided insight into the specific impact of prosthesis placement on overall satisfaction and quality of life. As this study is retrospective and relies on patient-reported outcomes, recall bias cannot be excluded and may have influenced the accuracy of some responses.

The questionnaire used was not a validated tool, although it was based on survey questions previously published in related studies. Furthermore, the survey answers were provided in multiple-choice format, leaving little room for nuance or detailed responses.

Future prospective, multicenter studies with larger cohorts are needed to validate these findings, enable multivariable analyses, and better define the role of TP in the long-term sexual health and quality of life of TC survivors.

## Conclusions

Overall satisfaction with TP placement is high, and patients who are dissatisfied are predominantly those who experience erectile dysfunction. The main concerns reported relate to the position, consistency, and size of the prosthesis. The frequency and quality of sexual encounters do not appear to be affected by TP placement. Preoperative counselling that addresses potential sexual function outcomes may help align patient expectations and further enhance overall satisfaction.

## Appendices

Appendix 1

**Table 10:** Questionnaire.

	Questions	Answers		
A. Decision-making factors				
1	You have agreed to the implantation of a testicular prosthesis. The "Normal" appearance of the organ is high importance for you?	Yes	No	I don't know
2	Did you have a steady partner when you decided to have a testicular prosthesis?	Yes	No	I don't know
3	Did having or Not having a partner play a role in your decision?	Yes	No	I don't know
B. Satisfaction				
1	Are you satisfied overall with your testicular prosthesis?	Yes	No	I don't know
2	You find that the position of the prosthesis	Very high	Appropriate	Very low
3	You find that the consistency of the prosthesis	Too firm	Appropriate	Too soft
4	You find that the size of the prosthesis	Too small	Appropriate	Too big
C. Effect on sexual life				
1	Do you find that your testicular prosthesis negatively affects your sexual desire (libido)?	Yes	No	I don't know
2	Do you find that having a testicular prosthesis negatively affects the quality of your erections?	Yes	No	I don't know
3	Do you find that you have problems with ejaculation due to the fact that you have a testicular prosthesis today?	Yes	No	I don't know
D. Short-term effect (within 1 year after testicular prosthesis insertion)				
1	Did the fact of having a testicular prosthesis decrease the frequency of sexual intercourse?	Yes	No	I don't know
2	Have you started testosterone replacement therapy?	Yes	No	I don't know
3	Did the fact of having a testicular prosthesis negatively affect the quality of sexual intercourse?	Yes	No	I don't know
4	Do you think the testicular prosthesis had an impact on your intimacy?	Yes	No	I don't know
E. Long-term effect (more than 1 year after testicular prosthesis insertion)				
1	Did the fact of having a testicular prosthesis decrease the frequency of sexual intercourse?	Yes	No	I don't know
2	Have you started testosterone replacement therapy?	Yes	No	I don't know
3	Did the fact of having a testicular prosthesis negatively affect the quality of sexual intercourse?	Yes	No	I don't know
4	Do you think the testicular prosthesis had an impact on your intimacy?	Yes	No	I don't know
